# Pain- and Fatigue-Related Functional and Structural Changes in Ankylosing Spondylitis: An fRMI Study

**DOI:** 10.3389/fmed.2020.00193

**Published:** 2020-05-15

**Authors:** Qi Liu, Zetao Liao, Yanli Zhang, Churong Lin, Bingjun He, Linkai Fang, Liudan Tu, Mingjing Zhao, Xinyu Wu, Jieruo Gu

**Affiliations:** ^1^Rheumatology Department of the Third Affiliated Hospital of Sun Yat-sen University, GuangZhou, China; ^2^Radiology Department of the Third Affiliated Hospital of Sun Yat-sen University, GuangZhou, China

**Keywords:** ankylosing spondylitis, pain, fatigue, fMRI, function connectivity, nodal properties, gray matter volumes

## Abstract

**Background:** Chronic pain and fatigue are two cardinal features of ankylosing spondylitis (AS) and how to effectively treat these conditions continues to be a challenge. The underlying mechanisms and the relationship between AS-related pain and fatigue remain poorly understood. The present study was conducted, therefore, to explore the brain functional and structural changes associated with pain and fatigue in AS.

**Methods:** A total of 65 AS patients (48 men and 17 women; 32.33 ± 8.6 years) and 53 age- and sex-matched controls were enrolled in the study. The patients underwent clinical assessment based on Total Back Pain scores, Fatigue Severity Scale, Bath Ankylosing Spondylitis Disease Activity Index, (BASDAI), high-sensitivity C-reactive Protein (hsCRP), erythrocyte sedimentation rate (ESR), and Beck Depression Inventory (BDI). Using 3T magnetic resonance imaging (3T-MRI), we analyzed the brain functional (connectivity and nodal properties) and structural (covariance and gray matter volumes) differences between AS patients and controls. Furthermore, we extracted the values of the significantly changed regions in the AS cohort and explored their association with pain and fatigue.

**Results:** In AS patients, there were functional and structural abnormalities distributed in the default mode network (DMN), salience network (SN), sensory/somatomotor network (SMN), dorsal attention network (DAN), task control network (TCN), and visual network, and some regions showed both types of changes. Among these, the functional connectivity (FC) between the left insula and medial prefrontal cortex, the betweenness centrality of the left medial prefrontal cortex and the gray matter volume of the right putamen tracked both pain and fatigue. In addition, pain was related to within-DMN FC disruption and nodal function / gray matter volumes changes in DMN, SN, and the visual network, while fatigue mainly involved the SMN, DAN, and TCN. Moreover, certain changes were also related to BASDAI and inflammation level.

**Conclusion:** This study offers new insights into understanding the neural mechanism of AS-related pain and fatigue, and could help to stratify patients based on the correlation features and ultimately move towards a personalized therapy.

## Introduction

Ankylosing spondylitis (AS) is an immune-mediated systemic inflammatory disease that mainly affects the spine and sacroiliac joint and is characterized as inflammatory low back pain (1). Inflammatory chronic back pain is one of the critical clinical criteria for the diagnosis of AS. Additionally, pain is a central element of the widely used clinical measure of AS activity known as Bath Ankylosing Spondylitis Disease Activity Index (BASDAI) (2), and evaluating pain relief is a recognized primary outcome in the assessment of treatment response in AS studies.

On the other hand, AS patients always suffer another major and frequent symptom—fatigue, which is reported in 50–70% patients (3) and closely related to disease severity, functional status, global well-being, and mental health. Conventional approaches to the treatment of fatigue, such as physical therapy and exercise programs, have proven to be unsatisfactory for the treatment of AS patients (4). Fatigue is also embedded in the BASDAI as a key indicator.

There have been a number of studies that have explored the relationship between AS-related pain and fatigue, but the results are inconsistent. Some researchers found AS-related fatigue to be strongly correlated with pain (5), but others suggested that fatigue could not reliably be controlled through pain relief (6). Besides, several studies have shown that pain could not explain the complete experience of fatigue (7, 8). A recent prospective study which recruited AS patients supported this disconnection between fatigue and pain, showing that pain could develop and worsen independently of tiredness, thus highlighting the necessity of understanding these symptoms independently (9).

In recent years, on the basis of functional MRI (fMRI) technique, there have been considerable evidence on the fatigue or pain-associated brain functional or structural alterations in chronic pain diseases (7, 10, 11). However, to date, no single study has explored both pain and fatigue-related central nervous system (CNS) changes in AS. In spite of growing information to support the view that a relationship exists between fatigue or pain and the brain, no consistent brain regions or change patterns have been reported. It should also be noted that most of the chronic back pain (CBP) studies recruited heterogeneous pain populations (i.e. idiopathic or mechanical back pain) (7, 12, 13). It is reasonable to believe that AS—as a unique systemic inflammation condition—causes specific changes in brain function and structure. Previous studies have suggested that chronic inflammation can contribute to predisposing individuals to neurological and neurodegenerative diseases through the cytokines released into the bloodstream (14). Furthermore, AS is a mixed pain state including both inflammatory and neuropathic components (15).

There have been only a few neuroimaging studies on AS (7, 10, 11, 16), and most of which had a small sample size (around 20 patients) and did not distinguish the effects of pain and fatigue. Furthermore, they focused either on the “pain matrix,” including the insula, the thalamus, the primary/secondary somatosensory cortex (SI/SII), the prefrontal cortex (PFC), and the anterior cingulate cortex (ACC) (15) or only on the default mode network (DMN), the salience network (SN), and the sensorimotor network (SMN) (13). However, recent studies have demonstrated that certain brain structure not belonging to the classical “pain matrix,” DMN, SN, or SMN may also participate in the central processing of fatigue or pain-related signals. Investigators have identified some brain alterations in vision-related brain regions in migraine (17), fibromyalgia (18), and knee osteoarthritis (19).

With these scientific problems in mind, the current study used a relatively large sample to: (i) identify the whole-brain network functional connectivity (FC) abnormalities associated with pain and fatigue in AS based on a validated, well-recognized human brain atlas (264 ROI) (20); (ii) characterize key regions associated with pain and fatigue based on brain network topology; and iii) on the basis of the identified functional alterations in i and ii, further analyze their underlying structural changes. Additionally, we explored the relationship between other clinical indexes (BASDAI score, ESR, CRP, and BDI) and our main findings.

## Methods and Materials

### Study Participants

A total of 65 AS patients (45 men and 20 women, aged 18-55) and 53 age- and gender-matched healthy controls (38 men and 15 women, aged 19-53) participated in the study. Our recruitment goal was to obtain a sample size of more than 50, as this would possess enough power to determine a moderately sized correlation at relatively conservative thresholds and is believed to be a sample size that performs well in receiver operating characteristic (ROI)-based unbiased functional connectivity analyses (21). Written informed consent was obtained from all subjects, and the study was approved by the Research Ethics Board of the Third Affiliated Hospital of Sun Yat-sen University. AS patients were recruited from the Department of Rheumatology of our hospital.

To be included in the study, AS patients had to meet the following criteria: (i) AS diagnosis that fulfilled the 1984 Modified New York Criteria (22), (ii) an average back pain score of ≥ 1 [on a 10-point scale] in the previous week; that is, we did not recruit patients totally free of pain. Fatigue severity measure was not used to determine suitability for inclusion; however, most patients (51 of 65) reported weariness to some extent; and (iii) did not receive any biologic agents (e.g. TNF blockers) for at least 2 months prior to the study.

The exclusion criteria for both patients and healthy controls were: (i) any contraindication for having an MRI; (ii) a history of psychiatric, neurologic, or metabolic conditions; (iii) major surgery within the past 2 years; or (iv) serious infection (associated with hospitalization or intravenous antibiotics) within 2 months of testing. Except for 1 control subject and 1 patient with AS, all participants were right-handed. The study was conducted strictly in accordance with the World Medical Association's Declaration of Helsinki and in line with relevant institutional and national guidelines.

### Clinical Assessment

All consenting subjects completed a set of clinical evaluation, which included total back pain (TBP) scores based on a scale of 0-10 (0 = totally free of pain, 10 = most serious pain imaginable) to evaluate the average degree of back pain experienced in the previous week. Fatigue was assessed using a modified 9-item, 10-point Fatigue Severity Scale (FSS), which is a unidimensional scale for assessing the influence of fatigue on daily living (23). Disease-specific variables included BASDAI (3), hsCRP, and ESR. Additionally, all study subjects completed a psychological questionnaire (BDI) (24).

### Neuroimaging Acquisition

Brain magnetic resonance image data were obtained with a 3T MRI scanner equipped with an 8-channel phased-array head coil (GE Medical Systems). All subjects were instructed to lie quietly in the supine position, and head motion was minimized with foam padding surrounding (outside) the head. Earplugs were used to attenuate the scanner noise. To exclude any structural abnormalities of the brain, we firstly obtained the conventional T1-weighted images (flip angle 70°, TR 200 ms, TE 2.78 ms, matrix 384 × 384, slice thickness 4.0 mm, 25 slices, voxel size 0.8 × 0.7 × 5 mm^3^) and fluid-attenuated inversion recovery (FLAIR sequence: flip angle 130°, TR 9000 ms, TE 93 ms, TI 2500 ms, matrix 256 × 256, slice thickness 4.0 mm, 25 slices, voxel size 1.0 × 1.0 × 4 mm^3^). Next, we obtained (i) a T1-weighted high resolution anatomical scan (3D IR-FSPGR sequence: flip angle 15°, 176 axial slices, TR 7.8 ms, TE 3 ms, 256 x 256 matrix, voxel size 1.0 × 1.0 × 1.0 mm^3^) for structural analysis, and (ii) an 8-minute T2-weighted resting-state fMRI scan (EPI sequence: 41 slices, TR 2000 ms, TE 30 ms, 64 x 64 matrix; voxel size 3 × 3 × 4 mm^3^) for functional analysis. During the fMRI scan, the patients and healthy controls were instructed to “open your eyes naturally, do not think about anything in particular and do not fall asleep.” All images underwent strict quality inspection by a neurologist and radiologist.

### Resting-State fMRI Pre-processing

fMRI data were preprocessed using SPM8 (https://www.fil.ion.ucl.ac.uk/spm/software/spm8/) and GRETNA 2.0 (http://www.nitrc.org/projects/gretna/) loaded on MATLAB R2014a (Mathworks, Sherborn, MA, USA), as previously described (25). In brief, we first discarded the first 10 volumes for equilibration purposes, and realigned the remaining 230 functional images to the first one. Then, we normalized the functional data based on T1-unified segmentation and smoothed these images with an 8 × 8 × 8 mm^3^ width Gaussian kernel. Next, the nuisances were regressed out from a mask comprising white matter, cerebrospinal fluid, and the whole brain, using the segmentation of the T1-weighted image, and 6 head motion parameters (3 translational plus 3 rotational), was also regressed. The temporal filter was in the range of 0.01–0.1 Hz. Finally, we performed scrubbing (26) to avoid motion artifact in the cross-regional correlation, in which all the frames showing excessive and sudden head motions were determined based on a predefined frame displacement (FD) threshold of 0.5 mm. We deleted all rejected frames from each time series, as well as the prior one and the 2 successive frames. The scrubbing procedure retained a mean proportion of 92.5% and 95.1% of slice frames in AS and healthy controls (HC) data, respectively.

### Functional Integration: Whole Brain Resting-State Connectivity

To explore the whole-brain inter-regional functional relationship, we constructed the brain network according to a set of 264-region-of-interest (ROI) atlas (covering 17 functionally parcellated networks) derived from resting-state functional connectivity meta-analyses (20, 27). The 264 nodes were input into the GRETNA as 10 mm diameter spheres. This atlas has been demonstrated to establish authentic network topologies (27). Utilizing a publicly available ROI atlas is conducive to the external validation of our results by other researchers and limits the possible bias in autonomously choosing independent components resulting from independent component analysis (ICA) using different datasets. We then created undirected and weighted Fisher z-transformed 264 × 264 bivariate correlation (Pearson's r) matrices for every subject based on the mean signal from all 264 nodes in the atlas. The strength of a FC between 2 nodes was treated as an edge: the Fisher-transformed (r to z) correlation between the average time series of 2 nodes. We then performed between-group FC analyses based on two-sample *t*-tests including age and sex as covariates. The significance threshold was defined at *p* > 0.05 with a multiple comparison correction of the false discovery rate (FDR).

### Functional Separation: Nodal Properties

Graph theoretical measures can reflect not only brain network functional integration, but functional separation based on nodal indices (28). On the basis of FC analyses, we obtained a 264 x 264 matrix for each participant. Next, we need to set the network sparsity (calculated as the ratio between the number of actual edges and all the possible edges in a given network). Because network sparsity has a fundamental impact on topological measures, reasonable network thresholding is necessary to guarantee appropriate network comparisons among different subjects and cost thresholds (29). We analyzed the nodal indices across a series of cost thresholds (0.01 ≤ cost ≤ 0.40, interval = 0.02) (30), and the resulting area under the curve (AUC) analysis was completed on node metrics across all sparsity thresholds. The AUC analysis reflects the curves of topological measure changes varied as the function of sparsity. The detailed analysis method was described using GRETNA toolbox.

Briefly, nodal degree centrality (DC) represents the number of connections to a given node and reflects a node's accessibility, and nodal betweenness centrality (BC) embodies the important roles of nodal communication across a given node as the bridge (31). On the basis of the AUC value of each node, we conducted comparisons to identify the differences in nodal degree and betweenness centrality between HC and AS patients. Group differences were tested based on two-sample *t*-test with FDR correction (*p* < 0.05). These analyses were conducted in GRETNA. Connectome figures were presented by BrainNet Viewer 1.7 (www.nitrc.org/projects/bnv/).

### Structural Analysis: Structural Covariance and Gray Matter Volume

In this part, we aimed to determine whether functional changes (if any) are derived from underlying structural abnormalities. It is well known that functional changes represent a state of transition over a relatively short period while structural changes need time to develop. Data were analyzed with the Computational Anatomy Toolbox (CAT12, http://dbm.neuro.uni-jena.de/cat12/) toolbox segment data pipeline and integrated into SPM12. Firstly, we spatially registered the 3DT1 images to the tissue probability maps (TPM) and segmented them into gray matter, white matter, and cerebrospinal fluid. We then performed the affine registration to the stereotactic MNI space using the ICBM152 space. Secondly, on the basis of the Jacobian determinants calculated from the normalization process, we completed high-dimensional DARTEL normalization and nonlinear modulation. Thirdly, we removed the noise and bias and normalized the intensities to avoid MRI inhomogeneities and noise. Finally, to reduce potential inaccuracies during the normalization process, we smoothed the GM image (8 × 8 × 8 mm^3^ Gaussian kernel). Moreover, we assessed the quality of the resulting images by visual check and analyzed the image quality index (utilizing the quality assurance (QA) framework in CAT 12, http://www.neuro.uni-jena.de/cat/), only including the participants with QA no less than B- in our final analyses. Two subjects did not pass the image quality control and were excluded in our final analysis.

Besides FC, brain networks can also be generated between any brain areas based on GM structural similarity. This was named as the gray matter (GM) structural covariance (SC) (32). We then used the significantly changed FC derived from functional analysis as ROIs and calculated their SC. Finally, we extracted the gray matter volumes (GMV) of the nodes showing altered BC or DC in graph theoretical analysis along with the nodes having significant FC, and compared the between-group SC and GMV differences.

### Statistical Analysis

Demographic data (age and gender), TBP, FSS, questionnaire scores (BDI), clinical index (hsCRP and ESR), and levels of mean relative motion recorded during the fMRI scan were compared between groups using independent sample *t*-tests. Significance for these tests was set at *p* < 0.05 (two-sided).

To examine the group differences in FC and nodal properties (BC/DC), we applied general linear models (GLMs) on the composite graph theoretical measures controlling for age and gender (two-sample *t*-test, significance *p* < 0.05, FDR corrected). To explore the group differences in SC and GMV, we used two-sample *t*-test based on the significant FCs and nodes found in functional analyses controlling for age, gender, and total intracranial volume (TIV) with FDR multiple correction (*p* < 0.05, two-sided).

Partial correlations between fMRI measures (FC, BC, DC, SC, and GMV) of the brain regions which showed group difference and clinical features (pain and fatigue) were analyzed in AS patients by means of Spearman's correlation or multiple linear regression models with stepwise elimination. Additionally, we examined the relationship between the above findings and other indexes (BASDAI, hsCRP, and BDI). The significance was set at *p* < 0.05 corrected with multiple comparisons of Bonferroni correction (p < 0.05). The age, gender, and education level of each subject were imported as covariates in the statistical analysis. All statistical analyses were conducted in SPSS version 17.0, and correlation graphs were generated using GraphPad Prism 8.

## Results

### Demographic and Clinical Data

Of the total 118 subjects recruited, 9 (8 AS+ 1 HC) were excluded because of head motion > 2 mm, and 5 (3 + 2) had MRI scans deemed unacceptable or acquired with different parameters, resulting in 54 AS patients and 50 HC being included in the final analysis. Table 1 lists the demographic and clinical data of all the subjects. There were no significant between-group differences in age (*t* = 0.04, *p* = 0.96, two-sample *t* test) or gender. All the AS patients reported a TBP score of at least 1, and 51 out of 65 AS patients reported experiencing fatigue (FSS) to some extent during the previous week. The group average TBP and FSS scores were 5.15 and 5.02 in AS patients, respectively. The AS group reported suffering from pain or fatigue for 7.8 (6.8) and 3.5 (4.3) years, respectively. At the time of enrolment, 38 AS patients were taking nonsteroidal anti-inflammatory drugs, which included Celebrex, Meloxicam, Diclofenac, and Ibuprofen. No patients had received other central analgesics or biologic agents in the past 2 months. To exclude possible determinants of AS-related pain, we conducted multiple regression analyses controlling for age and gender, where FSS, BASDAI, hsCRP, ESR, and BDI were selected as the dependent variables. Only BASDAI score was shown to be weakly associated with TBP score (*r* = 0.439, *p* = 0.012) and explained an approximate 30% variance in TBP scores. There was no significant correlation between TBP scores and age, sex, FSS scores, hsCRP, ESR, or BDI scores (*p* > 0.05) (Supplementary Table 1). Moreover, all of the patients had no other clear source of pain or fatigue (e.g. cancer or lumbar disc herniation) based on medical history or physical examination.

**Table 1:** Demographic and clinical data of AS patients and health controls.

	**AS patients (*n* = 54)**	**Health controls (*n* = 50)**	***p*-value**
Gender (male + female)	39 + 15	38 + 12	>0.05
Age (years)	32.33 ± 8.6	32.4 ± 9.2	>0.05
BASDAI	3.80 ± 1.31	N/A	
ESR (mm/hr)	7.52 ± 5.91	N/A	
hsCRP (mg/L)	5.89 ± 5.03	N/A	
TBP score	5.15 ± 1.95	N/A	
FSS score	5.02 ± 2.13	N/A	
Duration of back pain in years ± SD (range)	7.88 ± 6.89	N/A	
Duration of fatigue in years ± SD (range)	3.56 ± 4.31	N/A	
BDI (range)	6.8 (0-18)	2.1 (0-12)	0.001

*Data were expressed as mean ± SD. Abbreviations: BASDAI, Bath Ankylosing Spondylitis Disease Activity Index; ESR, erythrocyte sedimentation rate; hsCRP, high-sensitivity C-reactive protein; BDI, The Beck Depression Inventory*.

### Functional Integration: Whole Brain Resting-State Connectivity

**Figure 1:**
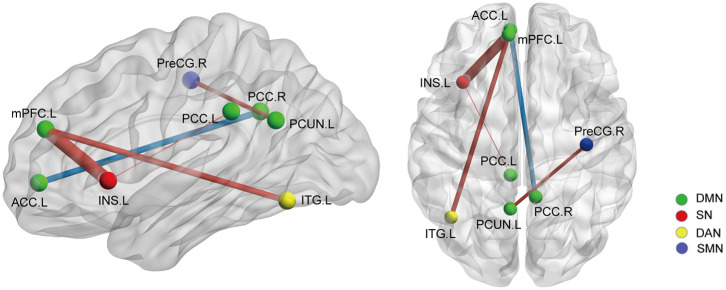
The whole network functional connectivity differences between the AS patients and HCs (*p* < 0.05, FDR corrected). Ball color indicates different brain networks. Red/blue line indicates the FC strengths in the AS cohort that were higher/lower (more/less positive correlation) than those in the HCs. The line width indicates the degree of differences (wider line means larger difference). DMN: default mode network; SN: salience network; SMN: sensory/somatomotor network; DAN: dorsal attention network.

**Figure 2:**
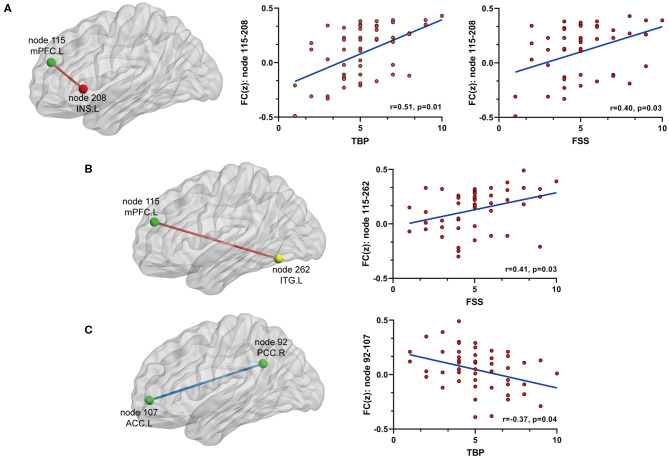
The relationship between significant FC and pain or fatigue as measured by TBP or FSS (*p* < 0.05, Bonferroni corrected): **(A)** FC between node 115 (mPFC) and node 208 (INS.L) was positively[[Inline Image]] associated with both pain and fatigue; **(B)** FC between node 115 (mPFC) and node 262 (ITG.L) was positively associated with fatigue; **(C)** FC between node 92 (PCC.R) and node 107 (ACC.L) was negatively associated with fatigue. mPFC, medial prefrontal cortex; INS.L, left insula; ITG.L, left inferior temporal cortex; PCC.R, right posterior cingulate cortex; ACC.L, left anterior cingulate cortex.

On the basis of the 264-ROI atlas, we found the following FCs to be significantly changed in AS patients compared with HC (*p* < 0.05, FDR corrected) (Supplementary Table 2 and Figure 1): (i) enhanced FC: node 208 (MNI:−35, 20, 0, left insula) and node 115 (−8, 48, 23, medial prefrontal cortex), node 208 and node 133 (−2,−35, 31, left posterior cingulate cortex), node 115 (−8, 48, 23, medial prefrontal cortex) and node 262 (−42,−60,−9, left inferior temporal gyrus), and node 88 (−7,−55, 27, left precuneus) and node 41(38, −17, 45, right precentral cortex); (ii) diminished FC: node 107 (−7, 51,−1, left anterior cingulate cortex) and node 92 (8,−48, 31, right posterior cingulate cortex). Of these nodes, nodes 88, 92, 107, 115, and 133 belong to the default mode network (DMN), node 208 to the salience network (SN), node 41 to the sensory/somatomotor network (SMN), and node 262 to the dorsal attention network (DAN). These findings indicate there are widespread cross- and within- network disruptions, mainly involving DMN followed by SN, SMN, and DAN in AS. Of the above-mentioned 5 significantly changed FCs, we found 3 to be correlated with TBP or FSS (*p* < 0.05, Bonferroni corrected): the FC between nodes 208 and 115 was correlated with both TBP and FSS; and the FC between nodes 115 and 262 with FSS, and the FC between nodes 107 and 92 were correlated with TBP (Figure 2).

### Functional Separation: Nodal Properties

**Figure 3:**
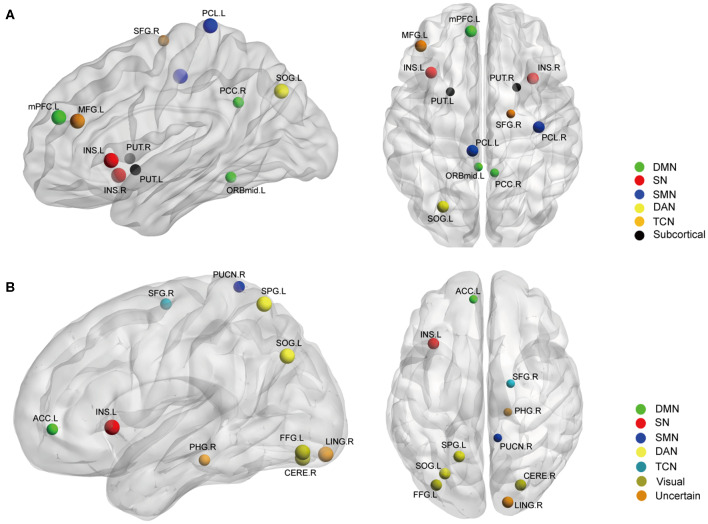
The nodal BC **(A)** and DC **(B)** differences between AS patients and HC (*p* < 0.05, FDR corrected). Ball color indicates different brain networks. Ball size indicates the difference direction (bigger ones: AS > HC, smaller ones: AS < HC). DMN, default mode network; SN, salience network; SMN, sensory/somatomotor network; DAN, dorsal attention network; TCN, fronto-parietal task control network.

**Figure 4:**
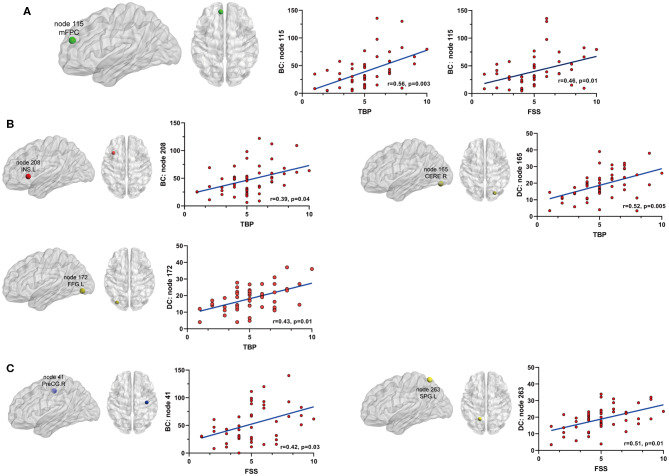
The relationship between significant nodal properties (BC and DC) and pain or fatigue as measured by TBP or FSS(*p* < 0.05, Bonferroni corrected): **(A)** BC of node 115 (mPFC) was positively associated with both pain and fatigue; **(B)** BC of node 208 (INS.L) and DC of nodes 165 (CERE.R) and 172 (FFG.L) were positively associated with fatigue; **(C)** BC of node 41 (PreCG.R) and DC of node 263 (SPG.L) were positively associated with fatigue. mPFC, medial prefrontal cortex; INS.L, left insula; CERE.R, right cerebellum; FFG.L, left fusiform gyrus; PreCG.R, right precentral cortex; SPG.L, left superior parietal gyrus.

Betweenness centrality (BC) is the fraction of all the shortest paths in the network that go through a given node and evaluates the contribution of a node based on the communication with other nodes. Nodes with higher BC possess higher efficiency in transferring information. Degree centrality (DC) refers to the number of ties a node has with other nodes and is another measure of node importance in the network. In our topological analyses, a dozen nodes were found to have altered BC or DC which was distributed in the DMN, SMN, DAN, TCN, SN, and visual and subcortical cortex (Table 2 and Figure 3). Further, the BC/DC of the nodes in the SN (node 208, left insula) and visual network (node 165, right cerebellum and node 172, left fusiform gyrus) were associated with AS-related pain, and the nodal properties of SMN (node 41, right precentral gyrus) and DAN (node 263, left superior parietal gyrus) were related to fatigue, while the BC of node 115 (medial prefrontal cortex) in the DMN was correlated to both TBP and FSS (*p* < 0.05, Bonferroni corrected, adjusted for age and sex) (Figure 4).

**Table 2:** Nodal topological differences between AS patients and HC.

Node^*^	**Peak MNI Coordinate**	**Approximate structure**	**Network**	**Direction**	***p*-value (FDR corrected)**
**Betweenness centrality**					
18	−7,−33, 72	Paracentral lobule_L	SMN	AS > HC	0.032
41	38,−17, 45	Precentral lobule_R	SMN	AS > HC	0.01
49	19,−8, 64	Superior frontal gyrus_R	TCN	AS < HC	0.022
92	8,−48, 31	Posterior cingulate _R	DMN	AS < HC	0.009
109	−3, 44,−9	Middle frontal gyrus_R	DMN	AS < HC	0.015
115	−8, 48, 23	Medial prefrontal cortex	DMN	AS > HC	0.013
188	−42, 38, 21	Middle frontal gyrus_L	TCN	AS > HC	0.03
208	−35, 20, 0	Insula_L	SN	AS > HC	0.046
211	34, 16,−8	Insula_R	SN	AS > HC	0.008
227	−22, 7,−5	Putamen_L	Subcortical	AS < HC	0.011
230	23, 10, 1	Putamen_R	Subcortical	AS < HC	0.023
260	−27,−71, 37	Superior occipital gyrus_L	DAN	AS > HC	0.045
**Degree centrality**					
7	17,−28,−17	Parahippocampal gyrus_R	Uncertain	AS < HC	0.011
22	10,−46, 73	Precuneus _R	SMN	AS < HC	0.025
49	19,−8, 64	Superior frontal gyrus_R	TCN	AS < HC	0.037
107	−7, 51,−1	Anterior cingulate_L	DMN	AS < HC	0.033
141	17,−91,−14	Lingual gyrus _R	Uncertain	AS > HC	0.015
165	26,−79,−16	Cerebelum_R	Visual	AS > HC	0.031
172	−33,−79,−13	Fusiform gyrus_L	Visual	AS > HC	0.02
208	−35, 20, 0	Insula_L	SN	AS > HC	0.005
260	−27,−71, 37	Superior occipital gyrus_L	DAN	AS > HC	0.043
263	−17,−59, 64	Superior parietal gyrus_L	DAN	AS > HC	0.015

* *Node number based on power 264-ROI atlas. DMN, default mode network; SN, salience/network; DAN, dorsal attention network; SMN, sensory/somatomotor network; TCN, fronto-parietal task control network*.

### Structural Analysis: Structural Covariance and Gray Matter Volume

**Figure 5:**
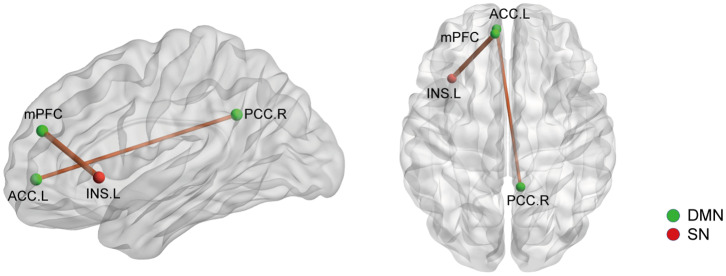
The structural covariance (SC) differences between the AS patients and HCs based on significant FC changes (*p* < 0.05, Bonferroni corrected). Ball color indicates different brain network. The line width indicates the degree of differences (wider line means larger difference). DMN, default mode network; SN, salience network.

We then selected the significantly changed edges and nodes found in the above functional study as ROIs. Firstly, we performed SC analysis based on our FC findings; that is, we calculated the SC between the nodes which had significantly different FC between AS patients and HCs (node 41, 88, 92, 107, 115, 133, 208, and 262) (also see Figure 1). We only found 2 SCs to be different in the AS group compared with HC (*p* < 0.05, FDR corrected): one was the increased SC between node 115 (MNI:−8,48,23, medial prefrontal cortex) and 208 (-35, 20, 0, left insula), and the other was also an increased SC between node 107 (-7, 51,−1, left anterior cingulate cortex) and node 92 (8,−48, 31, right posterior cingulate cortex) (Figure 5). Interestingly, the latter was in the opposite direction to FC change (decrease). In brief, AS patients have augmented DMN-SN and within-DMN SC.

**Figure 6:**
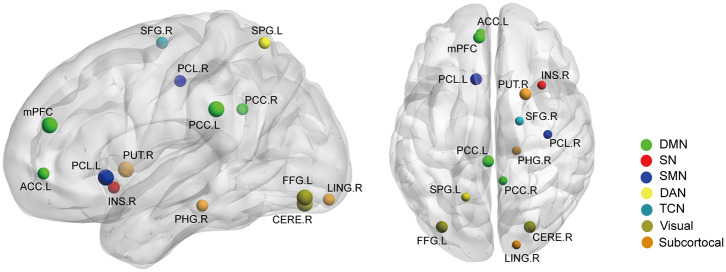
The gray matter volume (GMV) differences between the AS patients and HCs (*p* < 0.05, FDR corrected). Ball color indicates different brain networks. Ball size indicates the difference direction (bigger ones: AS > HC; smaller ones: AS < HC). DMN, default mode network; SN, salience network; SMN, sensory/somatomotor network; DAN, dorsal attention network; TCN, fronto-parietal task control network.

**Figure 7:**
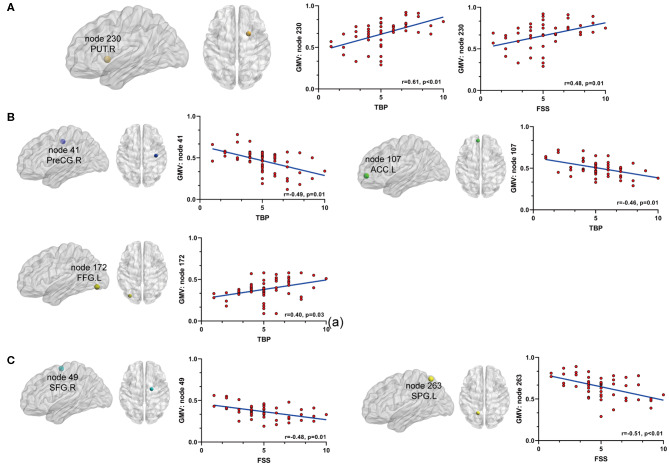
The relationship between significant GMV changes and pain or fatigue as measured by TBP or FSS (*p* < 0.05, Bonferroni corrected): **(A)** gray matter volume (GMV) of node 230 (PUT.R) was positively associated with both pain and fatigue; **(B)** GMV of node 41 (PreCG.R) and node 107 (ACC.L) was negatively associated with pain and GMV of node 172 (FFG.L) was positively associated with pain; **(C)** GMV of nodes 49 (SFG.R) and 263(SPG.L) was negatively associated with fatigue. PUT.R, right putamen; PreCG.R, right precentral cortex; ACC.L, left anterior cingulate cortex; FFG.L, left fusiform gyrus; SFG.R, right superior frontal gyrus; SPG.L, left superior parietal gyrus.

Secondly, we extracted the gray matter volumes from all the nodes showing different FC and nodal properties (see Figure 1 and Table 2) and made comparisons between the AS patients and HCs. Astonishingly, the majority of nodes also had significantly changed GM volumes in the AS patients compared to HCs (*p* < 0.05, FDR corrected) (Table 3 and Figure 6). Of these significant nodes, the GMV of node 230 (right putamen) tracked both TBP and FSS, the GMV of node 41 (right precentral gyrus) and 107 (left anterior cingulate cortex) was negatively associated with TBP, and the GMV of node 172 (left fusiform gyrus) was positively associated with TBP, while the GMV of node 49 (right superior frontal gyrus) and 263 (left superior parietal gyrus) was negatively correlated with FSS (*p* < 0.05, Bonferroni corrected) (Figure 7).

**Table 3:** Gray matter volume differences between the AS patients and HCs.

Node^*^	**Peak MNI**	**Approximate structure**	**Network**	**Direction**	*p*-value^**^ (FDR corrected)
7	17,−28,−17	Parahippocampal gyrus_R	Uncertain	AS < HC	0.032
18	−10,20,-3	Paracentral lobule_L	SMN	AS > HC	0.011
41	38,−17, 45	Precentral lobule_R	SMN	AS < HC	0.025
49	19,−8, 64	Superior frontal gyrus_R	TCN	AS < HC	0.03
92	8,−48, 31	Posterior cingulate _R	DMN	AS < HC	0.011
107	−7, 51,−1	Anterior cingulate_L	DMN	AS < HC	0.04
115	−8, 48, 23	Medial prefrontal cortex	DMN	AS > HC	0.021
133	−2,−35, 31	Posterior cingulate _L	DMN	AS > HC	0.018
141	17,−91,−14	Lingual gyrus _R	Uncertain	AS < HC	0.006
165	26,−79,−16	Cerebelum_R	Visual	AS > HC	0.023
172	−33,−79,−13	Fusiform gyrus_L	Visual	AS > HC	0.017
211	34, 16,−8	Insula_R	SN	AS < HC	0.023
230	23, 10, 1	Putamen_R	Subcortical	AS > HC	0.013
263	−17,−59, 64	Superior parietal gyrus_L	DAN	AS < HC	0.031

* *Node number based on power 264-ROI atlas*.

** *Corrected for total intracranial volume, age and sex. DMN, default mode network; SN, salience/network; DAN, dorsal attention network; SMN, sensory/somatomotor network; TCN, fronto-parietal task control network*.

### Secondary Analysis: Association With Other Clinical Indices

We also explored the association between the significant FC or nodes found in our primary analysis and the other clinic indices collected (BASDAI, hsCRP, ESR, and BDI). Our results were as follows: (i) the FC between nodes 208 and 115 was moderately correlated to BASDAI (*r* = 0.41, *p* = 0.02, Bonferroni corrected); (ii) the GMV of putamen was weakly associated with BASDAI, which did not pass the multiple correction (*r* = 0.34, *p* = 0.15, uncorrected); and (iii) the GMV decrease of node 115 (medial prefrontal cortex) was weakly associated with ESR, and also did not pass multiple correction (*r* = 0.31, *p* = 0.03, uncorrected) (Table 4 and Supplementary Figure 1).

**Table 4:** Secondary analysis: association with other clinic indices.

	**Approximate structure**	**Network**	**Clinic index**	***r* & *p* value**
FC: node 115 - 208	Left insula–medial prefrontal cortex	DMN-SN	BASDAI	*r* = 0.41, *p* = 0.031
GMV: node 230	Right putamen	Subcortical	BASDAI	*r* = 0.39, *p* = 0.03^*^
GMV: node 115	Medial prefrontal cortex	DMN	ESR	*r* = 0.37, *p* = 0.04^*^

* *Not corrected by multiple comparison. FC, functional connectivity; GMV, gray matter volume*.

### Supplementary Analysis: Effects of Head Motion, Depression Level and Treatment

We conducted additional tests to exclude the bias caused by head motion in our analyses. The mean relative framewise displacement in the AS patients was comparable to that in the HCs (0.13 ± 0.07 vs. 0.11 ± 0.03; *p* = 0.35, two-sample *t*-test). Moreover, our main findings remained largely unchanged after entering mean relative head motion as a covariate of no interest in our analyses.

Psychiatric disorders (including depression, which is a common complication in AS) are known to cause specific lesions in the brain. Although all the recruited patients were not clinically diagnosed as depressed or as suffering from other psychiatric conditions, the BDI of the AS patients was significantly higher than that of the HCs. However, there was no significant relationship observed between any of the above-mentioned FC, nodal properties, and GMV results with BDI scores. Therefore, it is unlikely that our main findings were confounded by depression.

Finally, to ensure our main findings were not biased according to treatment, we made a comparison between treated (*n* = 38) and treatment-naïve (*n* = 16) patients. There were no major between-group differences in any significant FC or nodes identified in our primary analysis (Supplementary Table 3).

## Discussion

To our knowledge, the current neuroimaging study has a relatively large sample size (particularly in rheumatic diseases) and for the first time demonstrates a prominent aberration in both function and structure associated with pain and fatigue in AS. Our key findings were: (i) there were multiple pain and fatigue-related FC changes in several key regions belonging to the DMN, SN, SSN, and DAN; (ii) AS-related pain mainly involved the DMN, SN, and visual networks, while fatigue was associated with the DMN, SMN, and DAN; (iii) some of the above changes could, at least in part, be explained by underlying structural alterations; and (iv) there were both distinct and overlapping functional and structural abnormalities correlated with pain and fatigue, reflecting the multifactorial nature of the two symptoms of AS at a neurobiological level.

Under normal conditions, the DMN and SN are anticorrelated (33). However, in many diseases, this anticorrelation is attenuated. The FC between the DMN and insula (an important region of SN) is changed in diabetic neuropathy (34), temporomandibular disease (35), and fibromyalgia (36). However, these findings have not been validated using a whole-brain design to construct a comprehensive explanation of how brain changes correlate with chronic pain or fatigue. Our findings validated the DMN-SN abnormalities. More importantly, the insula-mPFC FC was associated with clinical variables: it diminished after successful pain management in fibromyalgia (37) and increased when pain aggravated in CBP (38). We found the insula-mPFC FC in AS was associated with both pain and fatigue, which evidenced the importance of the insula and mPFC in the neural mechanisms of AS.

A region of DAN (left inferior temporal gyrus) also had increased connectivity to mPFC and interestingly, this FC tracked fatigue in AS. A key role of DAN is the top-down control of attention in response to predictable stimuli, which complements the ventral attention network's bottom-up control of attention to unexpected stimuli. An fMRI study on rheumatoid arthritis (RA) indicated that fatigue was related to mPFC-DAN FC (25). Psychology literatures have also documented the close relationship between fatigue and DAN (39, 40). We validated the above findings and further suggested that the left inferior temporal cortex rather than the whole DAN is connected to mPFC in AS.

Recent fMRI studies observed within-DMN disruptions in migraines (17), fibromyalgia (41), and diabetic neuropathic pain (34). We found the FC between left ACC and right PCC was the only weakened FC which was also associated with TBP in AS. Our results are somewhat different with other studies in terms of specific DMN regions and might reflect AS specific within-DMN changes rather than common abnormalities in chronic pain.

Brain topological analysis can identify nodes that might have a significant or contributing role in disease. Schrepf et al. demonstrated that RA patients with higher level inflammation exhibited changed properties in mPFC and inferior parietal lobule (25). Considering the importance of the PCC and insula in pain processing and modulation, and the characteristic altered communication between the DMN and SN in chronic pain, the association between pain and nodal properties of PCC and INS is not unexpected. An interesting finding is the nodal changes in the visual network, which was correlated to pain. We infer that this might be an adaptive neural remodelling as a response to persistent pain inputs via a cross-network process. Specifically, pain input is usually accompanied by other sensory stimulation, such as audition (42) and vision (43). As a consequence of the stronger influence of avoidance behaviours, AS sufferers usually pay attention to adjusting their body position with the help of their visual system. This finding further suggests an automatic adaptation mechanism via attention and motion regulation.

Previous studies have suggested that fatigue and pain exert different influence on the CNS: fatigue impacts more on the SMN and DAN than regions classically involved in pain perception (44). For instance, a majority of AS patients who received bio-agents experienced a transformation from a condition of pain to a fatigue prominent status, and TNFi treatment only recovered the pain-related brain regions (7); this is generally in agreement with our results. Additionally, our results stressed the important role of mPFC in both pain and fatigue. In brief, our findings suggest a hub disruption or a reorganization in the brain of AS patients, with generally different regions involved in pain or fatigue.

Our structural analyses suggested there were also underlying structural abnormalities in AS. The higher insula-mPFC SC might result from the increased insula-mPFC FC—a synchronous discharge of neurons induced the synapse formation between neurons. The increased SC between left ACC and right PCC might be a compensatory response resulting from disrupted within-DMN activity—the brain needs to enhance its structural synergy within the DMN to maintain stability in resting states.

GMV analyses further confirmed the roll of DMN and visual network in AS-related pain and the importance of DAN, SMN, and TCN in the pathogenesis of fatigue. First, GMV decrease of the primary somatosensory cortex might result from activity-dependent plasticity due to sensory loss, as evidenced by the mechanical and thermal hyposensitivity in the feet of the AS patients. Second, the GMV decrease of left ACC might be because of the preferential loss of inhibitory inter-neurons in this region, which could explain the increased pain effect mediated by the subgenual part of the ACC (45). Third, the visual network change further validated the above-mentioned adaptive neural remodelling hypothesis - visual inputs accompanying pain sensation enhance the visual network activity and, if this persists, adaptive GMV increase takes place. Finally, the importance of the putamen in pain and fatigue is remarkable. Interestingly, other neuroimaging studies on AS and RA also documented a similar relationship between the putamen GMV and fatigue (10, 25), suggesting this might be a generic pathway. From a biological perspective, the putamen plays a key role in reward response and a decrease in activity could result in a low level of motivation: a critical element of AS-related fatigue. We surmise that this GMV increase possibly reflects a compensatory process resulting from putamen activity decrease.

Our study has the following limitations: Firstly, the study design is cross-sectional; therefore, we cannot clarify the causality of the detected brain abnormalities. Alternatively, these changes might be the downstream signals. Regardless, our results could still be meaningful in that no recognized objective measure of pain and fatigue exists for AS at present. It is important to validate our results using the same cohorts based on a longitudinal design. Secondly, whether the correlates we found are AS-specific or generic in pain or fatigue is still unknown, as similarly designed studies in this field are scarce. Additional validation on distinct pain and fatigue disorders are therefore warranted. Thirdly, our observations might be confined to resting state. Attention networks (e.g. SN and TCN) are known to show enhanced activity when performing tasks or in an attention condition, so future attempts are necessary to replicate our findings using take-based paradigms.

## Conclusion

In conclusion, the present study is the first to clearly examine the brain in the pathophysiology of AS-related pain and fatigue. The level of pain and fatigue was correlated significantly to a series of functional and structural abnormalities in the brain, a few of which overlapped. Certain regions were also associated with other clinical characteristics, reflecting the multi-factor nature of these symptoms in AS.

Our study not only identified some initial targets for urgently-needed fatigue or pain-directed therapies, but also created an objective measure for neurobiologically stratifying AS patients. Additionally, applying machine-learning technology to an even larger sample can contribute to stratifying patients based on the correlation signature and subsequently move toward a personalized therapy. Meanwhile, neuromodulating the neural correlates may facilitate the verification of the causation of the selected brain structures and offers a potential therapy for specific symptoms. Pain and fatigue are the most predominant symptoms and the most heterogeneous of constructs; thus, clinicians need a more effective means of achieving mechanistically individualized treatments to help patients reach a better outcome.

## Data Availability Statement

The datasets generated for this study are available on request to the corresponding author.

## Ethics Statement

The studies involving human participants were reviewed and approved by the ethics committee of the third affiliated hospital of Sun Yat-sen University. The patients/participants provided their written informed consent to participate in this study. Written informed consent was obtained from the individual(s) for the publication of any potentially identifiable images or data included in this article.

## Author Contributions

All authors were involved in drafting the manuscript, revising it critically for important intellectual content, and in the final approval of the publication.

## Conflict of Interest

The authors declare that the research was conducted in the absence of any commercial or financial relationships that could be construed as a potential conflict of interest.
