# Supplementary material for: Pain- and Fatigue-Related Functional and Structural Changes in Ankylosing Spondylitis: An fRMI Study
Source: Front Med (Lausanne). 2020 May 15;7:193. doi: 10.3389/fmed.2020.00193 (PMC7242653; doi:10.3389/fmed.2020.00193)
Supplement: Supplementary file 1 [file Data_Sheet_1.docx]

Supplementary Material

# Supplementary Table 1 Multiple regression analysis for the TBP score in patients with ankylosing spondylitis*

| **Independent**  **variables** | **Dependent variable (TBP score)** | | | | |
| --- | --- | --- | --- | --- | --- |
|  | **Spearman’s correlation** | | **Multiple linear regression** | | |
|  | **r** | ***p*** | ***β*** | ***t*** | ***p*** |
| Age | -0.168 | 0.231 | -0.040 | -1.322 | 0.193 |
| Sex | 0.017 | 0.903 | 0.056 | 0.423 | 0.678 |
| FSS | 0.32 | 0.071 | 0.148 | 1.325 | 0.192 |
| BASDAI | 0.439 | 0.012 | 0.619 | 2.889 | 0.021 |
| hsCRP | -0.11 | 0.473 | -0.005 | -0.099 | 0.921 |
| ESR | -0.31 | 0.827 | -0.076 | -0.568 | 0.573 |
| BDI | 0.061 | 0.668 | 0.041 | 0.671 | 0.506 |

* Spearman’s correlation was used to investigate the correlation of total back pain (TBP) scores with other clinical variables. In multiple linear regression analysis, age and sex were arbitrarily entered into the model. Then, other variables including FSS scores, Bath Ankylosing Spondylitis Disease Activity Index (BASDAI) , hsCRP, ESR and BDI were considered in the model in a stepwise manner.

**Supplementary Table 2.** Whole-brain functional connectivity differences between AS patients and HC

| **Node*** | **Approximate structure** | **Network** | **AS** | **HC** | ***p*-value**  **FDR corrected** |
| --- | --- | --- | --- | --- | --- |
| 208 - 115 | left insula –  medial prefrontal cortex | DMN - SN | 0.09 ± 0.23 | -0.09 ± 0.15 | 0.007 |
| 208 - 133 | left insula –  left posterior cingulate cortex | DMN -SN | 0.06 ± 0.18 | 0.01 ± 0.20 | 0.041 |
| 115 - 262 | left middle frontal gyrus –  left inferior temporal gyrus | DMN – DAN | 0.14 ± 0.19 | 0.03 ± 0.17 | 0.019 |
| 41 - 88 | right precentral cortex –  left precuneus | DMN – SMN | 0.12 ± 0.22 | 0.05 ± 0.18 | 0.031 |
| 92 - 107 | right posterior cingulate cortex –  left anterior cingulate cortex | DMN – DMN | 0.04 ± 0.17 | 0.14 ± 0.21 | 0.023 |

*Node number based on power 264-ROI atlas. DMN: default mode network; SN: salience/network; DAN: dorsal attention network; SMN: sensory/somatomotor network.

**Supplementary Table 3.** Supplementary Analysis: Effects of Treatment

|  | **Treated**  **(n=38)** | **Not treated**  **(n=16)** | **t value** | ***p* value** |
| --- | --- | --- | --- | --- |
| **Functional Connectivity** | | | | |
| Node 115 - 208 | 0.09 ± 0.23 | 0.06 ± 0.28 | 0.48 | 0.21 |
| Node 115 - 262 | 0.15 ± 0.18 | 0.13 ± 0.21 | 0.31 | 0.76 |
| Node 92 - 107 | 0.04 ± 0.19 | 0.04 ± 0.19 | -0.002 | 0.99 |
| **Nodal Properties** | | | | |
| Node 115 | 38.1 ± 26.75 | 41.1 ± 32.11 | -1.12 | 0.16 |
| Node 208 | 46.2 ± 22.1 | 49.2 ± 34.2 | -0.39 | 0.07 |
| Node 165 | 17.7 ± 8.65 | 21.3 ± 6.91 | -1.78 | 0.52 |
| Node 172 | 17.11 ± 7.8 | 19.9 ± 5.55 | -1.03 | 0.11 |
| Node 41 | 48.51 ± 32.2 | 51.1 ± 29.8 | -1.07 | 0.29 |
| Node 263 | 17.81 ± 7.11 | 21.9 ± 7.11 | -1.92 | 0.78 |
| **Gray matter volumes** | | | | |
| Node 230 | 0.66 ± 0.15 | 0.65 ± 0.15 | 0.36 | 0.85 |
| Node 41 | 0.47 ± 0.12 | 0.43 ± 0.17 | 0.92 | 0.36 |
| Node 107 | 0.43 ± 0.17 | 0.39 ± 0.14 | 0.82 | 0.42 |
| Node 172 | 0.38 ± 0.12 | 0.37 ± 0.12 | 0.45 | 0.66 |
| Node 49 | 0.35 ± 0.07 | 0.38 ± 0.11 | -1.45 | 0.15 |
| Node 263 | 0.66 ± 0.13 | 0.67 ± 0.16 | 0.071 | 0.95 |

## Supplementary Figures


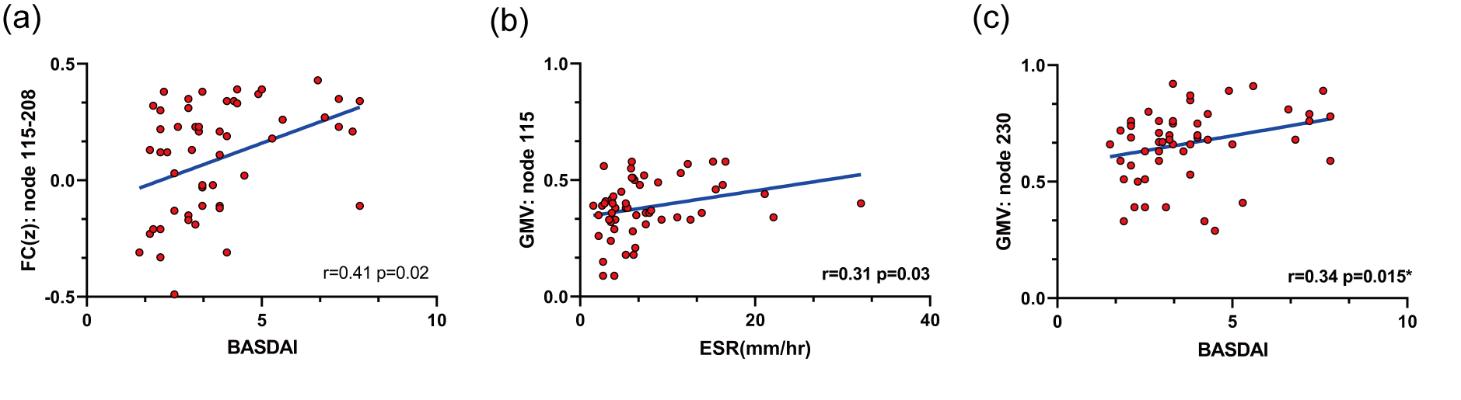


**Supplementary Figure 1.** (a)the FC between node 115(left medial prefrontal cortex) and 208(left insula) was positively correlated with BASDAI(p = 0.02, Bonferroni corrected); (b)the GMV of node 115(left medial prefrontal cortex) was positively correlated with ESR(p = 0.03, uncorrected); (c)the GMV of node 230(right putamen) was positively correlated with BASDAI(p = 0.015, uncorrected). FC: functional connectivity, GMV: gray matter volume
